# Optimizing colorectal cancer screening through quality circles of primary care physicians: a cluster randomized controlled trial

**DOI:** 10.3389/ijph.2026.1608462

**Published:** 2026-05-20

**Authors:** Tamara Scharf, Marc-Andrea Janggen, Yonas Martin, Julian Jakob, Kali Tal, Nikola Biller-Andorno, Jean-Luc Bulliard, Kevin Selby, Reto Auer, Adrian Rohrbasser

**Affiliations:** 1 Institute of Primary Healthcare (BIHAM), University of Bern, Bern, Switzerland; 2 Graduate School of health Sciences, University of Bern, Bern, Switzerland; 3 Department of Infectious Diseases, Bern University Hospital, University of Bern, Bern, Switzerland; 4 Department of Paediatrics, University Hospital Bern, Inselspital, Bern, Switzerland; 5 Institute for Biomedical Ethics and History of Medicine (IBME), UZH, Zürich, Switzerland; 6 Center for Primary Care and Public Health (Unisanté), Lausanne, Switzerland; 7 Medbase, Wil, Switzerland

**Keywords:** colonoscopy, colorectal cancer, fecal occult blood testing, pragmatic trial, primary care

## Abstract

**Objectives:**

Colorectal cancer (CRC) screening rates are low in Switzerland. This study tested whether a PCP intervention—training sessions and performance feedback within quality circles (QC) increased CRC screening rates.

**Methods:**

A pragmatic randomized controlled trial was conducted in Switzerland (2018–2021) with PCP in QC. The intervention included three training sessions, shared decision-making materials, and performance feedback based on 40 consecutive patients per PCP. The primary outcome was the difference in CRC screening rates between the intervention and control group after 12 months.

**Results:**

Of 120 invited QC, nine participated (5 intervention, 4 control). A total of 63 PCPs (32 intervention, 31 control) collected data on 2,112 patients (1,130 intervention, 982 control; mean age 61.5, 53% women). Analysis clustered by PCP and QC showed screening rate was 58% in the intervention group vs. 42% in controls (OR1.98; 95% CI:1.14–3.42). Screening rates in the intervention group increased from 55% to 57.9% (absolute increase: 2.9%; 95% CI:1.1%–6.9%; OR1.29; 95% CI:1.08–1.55, p < 0.01).

**Conclusion:**

Training sessions and performance feedback in QC increased screening rates, but few QCs chose to participate.

**Clinical Trial Registration:**

ClinicalTrials.gov, identifier NCT03510858.

## Introduction

### Colorectal cancer screening in primary care

International guidelines recommend several CRC screening methods for average-risk patients between 50 and 75 years old: colonoscopy every 10 years or fecal occult blood test (FOBT) every 2 years [1–3]. The European Commission issued a recommendation to offer breast cancer, cervical cancer and CRC screening to 90% of the population by 2025 [4]. If offered both screening options, patients who agree to CRC screening divide nearly equally between colonoscopy and FOBT [5, 6], but studies show most patients in Switzerland are screened with colonoscopy [5].

Screening rates in Swiss primary care are close to 50%, and almost all screening tests are performed using colonoscopy. However, our cross-sectional study among Swiss Primary Care Physicians (PCP) and an international randomized controlled trial from the US both suggest that screening rates could reach up to 70% if PCP offered patients both options and helped them make the decision that best suited their values and preferences. [7–9] Interventions that encourage PCP to promote both FOBT and colonoscopy as valid choices have been tested with varying success. Most of these interventions have focused on educating patients and providing PCP with communication tools, but few have focused on changing the prescription practices of PCP [10].

We hypothesized that providing PCP with the latest evidence on CRC screening, collecting data on their screening practices, encouraging them to engage in shared decision-making for CRC screening decisions supported by communication tools such as decision aids and providing feedback on their performance would raise their patients’ overall screening rate and increase the number of prescriptions for FOBT screening. We collaborated with physicians and patients to develop such a training program and materials that would encourage them to promote both screening methods [8] and decided to test it in quality circles (QC) because 80% of PCP in Switzerland attend QC [11].

QC are small groups of 8–12 PCP who regularly meet to reflect on and improve their standard practice. The participating PCP either work together in a group practice or have individual practices in the same region, and therefore treat similar patients. In QC sessions, PCP review clinical cases and current literature. Based on their local needs, QCs choose key areas for improvement and design improvement measures to change PCP practices [12].

We set out to test whether our intervention based on training sessions and performance feedback resulted in higher CRC screening rates in a cluster randomized controlled trial among PCP in QC.

## Methods

### Trial design and randomization

This was a pragmatic, open-label, cluster randomized controlled clinical trial, starting in April 2018 and ending in January 2021. To minimize the risk of crossover, we randomized at the level of the QC, ensuring that PCP working in the same practice were assigned to the same group. We enrolled all QCs in two waves: one in 2018 and one in 2019. Each QC was assigned a number, and this was communicated to the clinical trial unit (CTU) of the University of Bern. The CTU then used the software R version 3.4.0 to randomly allocate practices in a 1:1 ratio, without stratification, with a block size of two. The results of the allocation were then communicated to the research team.

### Participants and setting

In Switzerland, 52% of all PCP belong to a physician network that provides primary healthcare services and organizes QCs to improve the quality of care [13]. We thus decided to go through physician networks to contact enough Swiss PCP and their QCs for the trial. We invited QC moderators to participate in our trial via email invitations distributed to physician networks. We enrolled QCs from January 2018 to March 2019 until we reached the necessary number of QCs determined through our sample size calculation. In the German-speaking study regions of Switzerland where our QCs operated, CRC screening was opportunistic at that time. Screening occurs when patients interact with the healthcare system, most often during a consultation with a PCP. Mandatory health insurance reimburses CRC screening tests, including FOBT and colonoscopy. Screening is usually initiated by PCP, who either perform FOBT themselves or refer patients to specialists for colonoscopy. According to cantonal regulations, PCP or specialists bill associated costs directly to insurance companies. The other language regions were not contacted because language posed a barrier. The intervention involved extensive communication about the training program, and our research team was not trained to deliver this in French and/or Italian.

All German-speaking QCs comprised of PCP were eligible for inclusion. There were no exclusion criteria. Our invitation asked representatives of interested QCs to contact us directly. Once contacted, we invited the moderator to confirm the group’s interest by participating in a written survey. The survey requested information on the moderators’ credentials, meeting frequency and group size, documentation methods, additional tools regularly used to facilitate meetings, the number of PCP who regularly participate, and funding received (see Supplementary Appendix “moderator survey”). If the survey was filled out by the individual QC participant, we considered this as consent of the individual. We also affirmed that the QC had no financial interest in prescribing a specific CRC screening method. Once we received the completed survey sheets from one QC, we included the QC into the randomization process. We then informed moderators about their allocation to the intervention or control group. Immediately after randomization, we invited QCs allocated to the intervention group to schedule a meeting for the first intervention visit. We invited QCs in the control group to schedule a meeting a year later (See Supplementary Appendix “Timeline intervention and control”).

### Intervention

The intervention included a training program and data collection sheets. Our research team developed and refined the sheets based on feedback from physicians. We retested and adapted them to meet the needs of PCP in our pilot intervention in a Swiss QC and then used these materials in a trial that involved Swiss PCP [8].

For this study, the training program consisted of three visits to the intervention QCs. Trained study team members visited QC meetings, collected data through questionnaires, delivered the intervention through PowerPoint presentations and shared the materials physically as printouts. The intervention period lasted approximately 1 year, with each QC meeting occurring at four-month intervals (although these varied slightly as QCs were on different schedules). We began collecting data in March 2018, at the first intervention QC, and stopped collecting data after our second visit to the last control QC in January 2021. Details of the QC visits are described below; intervention QC visits sometimes ran parallel to control QC visits.

#### First visit - introduction to intervention and to data collection

If the QC moderator anticipated that the material for the initial visit might be too extensive and informed us in advance, we divided the visit into two separate sessions held no more than 1 month apart. The content remained unchanged.

At this first meeting, we shared the current guidelines for CRC screening, in the form of a 2-page structured evidence summary we developed earlier, which included colonoscopy and FOBT (Supplementary Appendix “Decision box”). We explained how to use the communication tools: a patient decision aid (a 20-page booklet) on CRC screening and a plasticized 4-page abridged version of the booklet to assist PCP in discussing CRC screening with patients during clinical visits (Supplementary Appendix “Decision Board”). We suggested ways to incorporate these tools into daily practice and allowed the QC to discuss how they could adapt them for their individual patient consultations.

Then we introduced the data collection sheets (see Supplementary Appendix “Data collection form”) that we developed with PCP and tested in previous studies [8, 14, 15]. The form was based on an algorithm (Supplementary Appendix “Flow Chart Data collecting”) designed to fit into a PCP’s routine. PCP were to use the data sheet to systematically report on 40 consecutive, non-urgent face-to-face consultations with eligible patients for visits longer than 5 min. All patients aged 50 to 75 seen during the data collection period were eligible for inclusion. Since we only collected data on age, sex, general information about CRC testing, and details on the consultation, the data could not be linked back to an individual patient. We showed PCP how to use the sheets. The research team provided PCP unfamiliar with FOBT a sample kit. We strongly encouraged PCP to deliver immunological FOBT (iFOBT or FIT) rather than guaiac-based FOBT (gFOBT). All PCP received two copies of the data collection sheet, which contained enough room to record 40 patient consultations. We also gave PCP an explanation sheet that contained a more detailed guide on how to use the sheet (see Supplementary Appendix “Data collection explanation”). The research group set a deadline of 2 months for collecting the data.

#### Data collection and visualization after the first visit

After the introduction to the intervention, PCPs began collecting data on patient consultations, filling up two sheets of the data collection form (20 patients per page), and returned the sheets to our team by email or post. PCP reported the number of weeks it took them to collect data on all 40 patients. They collected birth year, sex, and information about previous CRC testing (no previous testing, colonoscopy within the last 10 years, colonoscopy more than 10 years ago, FOBT within the past 2 years, FOBT more than 2 years ago, other tests, and unknown). To simplify data collection, the form did not differentiate between gFOBT and FIT. PCP who felt overburdened by collecting data on consecutive patients could include the first two eligible patients they saw per half-day of work for up to 2 months.

PCP were given a protocol for filling out the data sheet based on their consultations (Supplementary Appendix “Data collection overview”). If patients had been tested within the recommended intervals, PCP collected no more data. If patients had never been tested, PCP reported if CRC screening was contra-indicated (life expectancy <5 years, current severe condition, or other). If patients were eligible for a discussion about CRC screening, PCP reported the discussion or explained why they did not discuss screening. Reasons for not discussing could include: visit was not appropriate for discussing screening; it had already been discussed; the patient had already been seen during data collection; or other medical reasons. If a discussion took place, PCP noted the presence of potential CRC symptoms (bloody stools, abdominal pain, weight loss, change in bowel habits, others), risk factors for CRC (personal history of CRC or polyps, family history of CRC or polyps, personal history of Crohn’s disease or ulcerative colitis, or other), possible patient refusal, and reason(s) for refusal (no reason; patient did not feel concerned, feared side effects or complications, faced financial barriers, or other reasons). Finally, PCP indicated if patients planned a test and their chosen method (no screening planned, colonoscopy, FOBT, or other). To make it easier for PCPs to use the forms and to standardize data collection between individual physicians and QCs, we gave PCPs examples of completed forms based on short clinical vignettes.

After we received all the sheets, we analyzed this first set of data of the intervention group and generated descriptive graphs that illustrated patients’ CRC screening rate before and after the consultation, CRC screening discussion rates, and screening refusal rates (see Supplementary Appendix “Example of individual performance feedback for PCP of a QC”).

#### Second visit – workshop

After 2 months, we visited the QCs again and presented our results as performance feedback to each PCP in the QC group through a PowerPoint presentation (see Supplementary Appendix “Example of individual performance feedback for PCP of a QC”). In our summaries for the QC, we blinded PCP identity by assigning each a random number and unblinded the results if the QC agreed. Our team encouraged the QC to discuss and reflect on the results. We asked if they had performed as they expected, and if not, why their performance was unexpected and why they thought some PCP performed better than others. We asked the QC as a group if they wanted to improve their performance, and how they thought they could accomplish this. The group discussed improvement strategies and adapted the intervention to their local context and needs. We informed them that the second round of data collection (follow-up) would begin a year after this second visit, and then we would know if the improvement strategy the QC chose had changed their performance.

#### Third visit – follow-up

The trial follow-up period ended in January 2021. One year after the second visit, we mailed another two data collection sheets to the QCs. PCP received instructions to re-collect data from 40 patient consultations over a period of up to 2 months, employing the identical methods, to form a second data set, and then send the completed sheets to our research group. We analyzed the data in the same way, generating similar descriptive graphs to illustrate individual PCP performance. In the new graphics, we included the results from the previous year. Once our team received all the data collection sheets, we scheduled the third visit with the QCs. There, we held another workshop like that of the second visit and showed changes in PCP performance.

### Control

**FIGURE 1:**
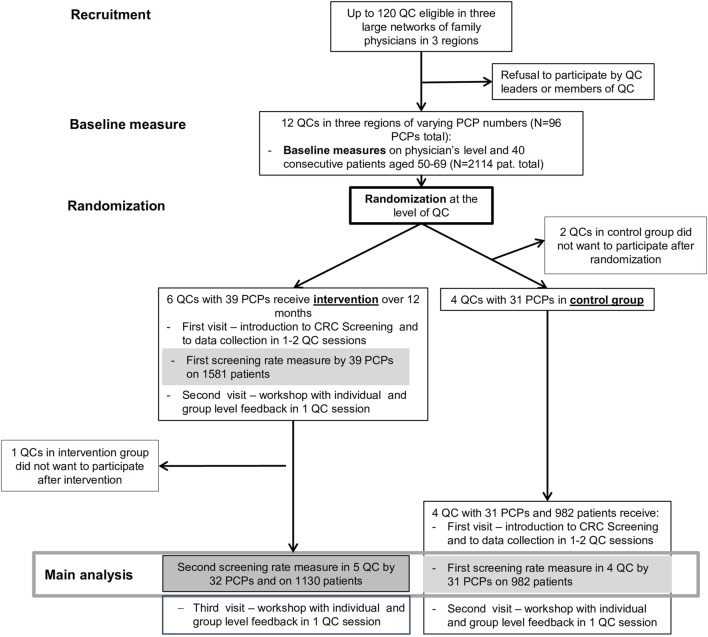
Inclusion and randomization flowchart (Optimizing colorectal cancer screening through quality circles of primary care physicians, Bern, Switzerland 2021).

Approximately 1 year after the initial visits of the intervention group, and at the same time as their third visits, we conducted the first visits to the control QCs. These control QCs then collected their first data on PCP consultations. After these data had been gathered and examined, we organized a second visit with individual and group level feedback in one QC session (see Figure 1).

### Outcome measures

The primary outcome was the difference between intervention and control groups in the proportion of eligible patients who were up to date with CRC testing at follow-up. This included any self-reported or documented colonoscopy performed within the last 10 years, or FOBT within the last 2 years before the patient consulted with the PCP. Secondary outcomes were 1) proportion of PCP whose data showed at least one patient with previous prescribed FOBT, 2) rate at which PCP discussed CRC screening with eligible patients (patients not previously tested within recommended intervals and without contra-indication for screening), and 3) the proportion of patients who were previously tested, or who planned to be tested after discussion, or who refused testing after discussion. We included missing data on the outcome as negative (not screened/not up-to-date).

### Statistical analysis and sample size calculation

We used descriptive statistics to report PCP screening data: the proportion of PCP who consulted with patients eligible for CRC screening vs. those who were not eligible. We reported whether testing had been discussed, refusal rates, and the chosen testing method after discussion. Our analyses followed a modified intention-to-treat principle. We included all patients recruited by randomized PCP in QCs who provided outcome data; however, any potentially enrolled patients assigned to PCP in QCs who withdrew before patient enrolment could not be included due to the absence of patient-specific outcome data.

Before starting our study, we calculated the sample size needed to detect significant differences between groups. We estimated a 9% increase in the proportion of patients who would opt for screening in the intervention group compared to the control with 25% of patients expected to have already been screened in primary care in Switzerland [16] and 5% refusing screening [17]. We assumed a hierarchical clustering structure with patients nested within PCP and PCP nested within QCs. We therefore estimated an intraclass correlation coefficient (ICC) of 0.05 at the PCP level (accounting for correlation of patient outcomes within PCP) and 0.005 at the QC level (accounting for correlation among PCP within the same QC) [18–20]. Based on these assumptions, enrolling 12 QCs with 8 PCP each and 40 patients per PCP would provide 80% power to detect a 9% absolute difference in screening uptake (from 40% [17] to 49%) [21].

For the primary outcome, we estimated each PCP’s rate of patients who were up to date with testing 1 year after our first data collection compared to the control groups first data collection, based on the reports of tests conducted on their 40 patients at the start of the consultation as a binary outcome at the patient level (up to date vs. not). For secondary outcomes, we used the same data to estimate each PCP’s overall FOBT prescription rate at the start of and after the discussion. We applied hierarchical multivariate logistic regression models, based on grouping of PCP by QC.

### Sensitivity analysis

We ran a sensitivity analysis to compare the first data collection from the intervention group to the second data collection a year later. Additionally, we used multiple imputation and mean substitution for the missing baseline characteristics of physicians and patient screening rates, using physicians baseline characteristics and patient sex and age as unconditional variables. We then ran hierarchical multivariate logistic regression models again.

Our research group conducted all statistical analyses on Stata version 15.1 (StataCorp). We considered a 2-sided P <0 .05 to be statistically significant. Our analysis began in July 2021 and ended in December 2021.

### Deviation from the protocol

In our registered study protocol, we planned to test for additional secondary outcomes: 1) the intention to prescribe tests to screen for CRC and 2) the intention to prescribe colonoscopy vs. FOBT within the next 6 months [22]. We intended to use a questionnaire to gather data on physicians’ prescribing intentions, but this approach proved impractical because many physicians misunderstood the questionnaire or did not return it. We did not collect enough completed questionnaires to analyze the intention outcomes, so we removed these outcomes from our main analysis. The trial was originally scheduled to last until September 2020 but the COVID-19 pandemic delayed QC meetings for several months, pushing the trial end date back by 4 months to January 2021. The sensitivity analysis comparing the first data collection from the intervention group to the second data collection a year later was not initially planned. This analysis was added during the process, because we were concerned about potential selection bias in PCPs collecting data.

### Patient and public involvement

We developed all our intervention materials in collaboration with a patient focus group consisting of five women and three men aged 50 to 75. They helped us focus on patient priorities, and preferences. We designed our own data collection chart to record patients’ refusals to be screened, including their reasons for doing so.

## Results

### Participant flow

The trial began in January 2018 and ran until January 2021. Of the 120 QC we deemed eligible, 12 (10%) responded to our invitation and were randomized at the practice level (see Figure 1). After three QC dropouts (two control groups decided not to participate, one intervention group was lost to follow-up) 9 QC (8%) remained, totaling 32 PCP, who collected data on 1,130 patients in the intervention group; 31 PCP collected data on 982 patients in the control group (see Figure 1).

### Baseline characteristics

Baseline characteristics of PCP and patients are reported in Table 1. The mean age category of PCP was 40–49 years; 41% were women (See Table 1).

**TABLE 1:** Characteristics of participating primary care physicians and their patients (Optimizing colorectal cancer screening through quality circles of primary care physicians, Bern, Switzerland 2021).

PCP characteristics	Intervention	Control	p-value
Quality circles, N	5	4	​
PCP, N	32	31	0.441
PCP per quality circle, median (range)	8 (3–9)	7 (6–10)	0.459
Age category, n (%)	​	​	0.274
20–29	3 (9)	0	​
30–39	10 (31)	6 (19)	​
40–49	8 (25)	13 (42)	​
50–59	7 (22)	7 (23)	​
>60	2 (6)	2 (7)	​
Age category missing	2 (6)	3 (10)	​
Female sex, n (%)	13 (41)	13 (42)	0.728
Sex missing	1 (3)	3 (10)	​
Area of practice, n (%)			
Urban	31 (97)	23 (74)	0.049
Rural	0	4 (13)	​
Missing	1 (3)	4 (13)	​

A total of 63 PCP collected data in our first data collection round with up to 40 patient consultations. Mean patient age was 62 and 61 years in the intervention and control group, respectively; 45% and 62% were women in the intervention and control group (see Table 2).

**TABLE 2:** Characteristics and screening outcomes of patients in intervention and control Groups on the first data collection round (Optimizing colorectal cancer screening through quality circles of primary care physicians, Bern, Switzerland 2021).

Data collection results	Intervention	Control
Total number of patients, N	1,130	982
Age, mean (sd)	62 (7.3)	61 (7.3)
Missing, n (%)	0	0
Female sex, n (%)	507 (45)	607 (62)
Missing, n (%)	0	1 (0.1)
Up to date with CRC screening (colonoscopy <10 years or FOBT <2 years before clinical visit), n (%)	655 (58)	416 (42)
Missing CRC screening, n (%)	63 (6)	70 (7)
Tested with colonoscopy within the last 10 years, n (%)	587 (52)	375 (38)
Tested with FOBT within the last 2 years, n (%)	60 (5)	30 (3)
Other tests to date, n (%)	8 (0.7)	11 (1.1)
Screening not up to date, or missing information, n (%)	412 (37)	496 (51)
Contraindications for screening, n (%)		
Life expectancy <5 years	14 (1)	17 (2)
Current severe disease	19 (2)	31 (3)
Other^a^	17 (2)	6 (1)
CRC symptoms	13 (1)	10 (1)
CRC risk factors	16 (1)	20 (2)
Was screening discussed or not during consultation	399 (100)	484 (100)
Screening was discussed during consultation	207 (52)	293 (61)
Not discussed during consultation because, n (%)		
- Patient/situation not suited for discussion	122 (31)	84 (17)
- Discussion already took place	20 (5)	12 (3)
- Patient already included (repeat visit during data collection)	3 (1)	13 (3)
- Other^a^ - Missing	47 (12) 8 (2)	82 (17) 11 (2)
Data on whether a testing decision was made	204 (100)	297 (100)
Decided for testing	149 (72)	219 (75)
Decided against testing because, n (%)		
- Did not feel concerned about CRC	13 (6)	39 (13)
- Fear of adverse effects of test	4 (2)	4 (1)
- Financial considerations	3 (1)	2 (1)
- No reason reported	12 (6)	13 (4)
- Other^a^	23 (11)	20 (7)
Data on decision collected	149 (100)	217 (100)
Planned test, n (%)		
- Colonoscopy	57 (38)	88 (41)
- FOBT	59 (40)	81 (37)
- Other	0	1 (1)
Decision deferred	18 (12)	43 (20)
No decision reported	15 (10)	4 (2)

^a^ Other/missing: PCP, could select this if no other option fit the case. No further specification was requested.

### Primary outcomes

**FIGURE 2:**
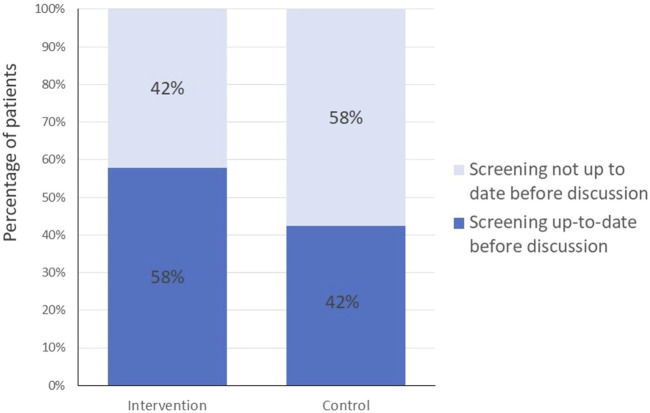
Primary outcome: colorectal cancer screening status of patients before discussions; intervention group data 1 year after intervention (Optimizing colorectal cancer screening through quality circles of primary care physicians, Bern, Switzerland 2021).

After 1 year, the proportion of patients up to date with CRC screening was higher at follow-up in the intervention group than in the baseline data collection in the control group (58% vs. 42%); the absolute, unadjusted risk difference was 15.5% (95%CI:11.3%–19.7%) and the OR was 1.98, (95% CI 1.14 to 3.42, p 0.02) controlled for the hierarchy of PCP and QC and an OR 1.74, (95% CI 1.00 to 3.02, p 0.05) adjusted for age, gender and location of the physicians and age and gender of patients, See Table 3; Figure 2 for details.

**TABLE 3:** Main outcomes comparing the second data collection in the intervention with the first in the control group (Optimizing colorectal cancer screening through quality circles of primary care physicians, Bern, Switzerland 2021).

Outcomes	Intervention N (%)	Control N (%)	Unadjusted OR (95% CI)	Adjusted OR (95% CI)
Main outcome				
Number of patients who had been tested for CRC before the discussion	655 (58)	416 (42)	1.98 (1.14–3.42)	1.74 (1.00–3.02)
Secondary outcomes				
Number of PCP who had at least one patient screened with FOBT	12 (37.5)	11 (35.5)	1.09 (0.39–3.05)	1.75, (0.46–6.62)
Number of patients tested for CRC before the intervention or who decided to be tested at the end of the consultation	771 (68)	586 (60)	1.37 (0.81–2.31)	1.31 (0.69–2.48)

### Secondary outcomes

A similar proportion of PCP in the intervention and control groups offered at least one FOBT to their patients: 37.5% (n = 12/32) vs. 35.5% (n = 11/31). Absolute Risk difference was 2%, OR 1.09 (95% CI: 0.39 to 3.05, p 0.87), adjusted for age, gender and location of PCP resulting in an OR of 1.75, (95% CI: 0.46 to 6.62, p 0.41) See Table 3 for details.

There was a trend towards fewer PCP discussing CRC screening with eligible patients in the intervention group (207; 52%) than in the control group (293; 61%), albeit not reaching statistical significance. Absolute risk difference was 9%; OR 0.48 (95% CI: 0.19 to 1.49, p 0.19), OR 0.42 (95% CI: 0.12 to 1.45, p 0.17) adjusted for age, gender and location of the physicians and age and gender of patients.

**FIGURE 3:**
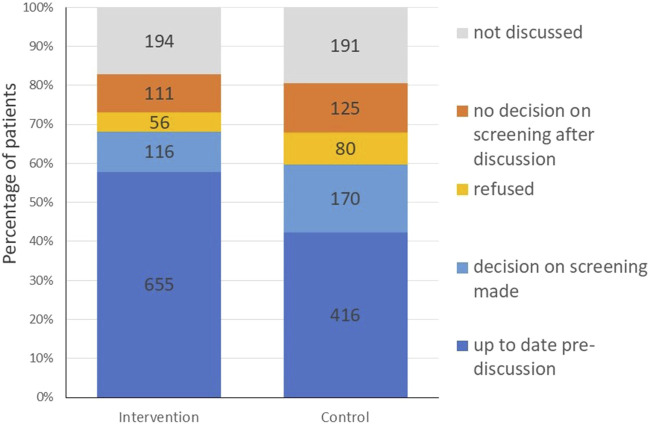
Secondary outcomes: colorectal cancer screening status of patients before and after discussion; intervention group data 1 year after intervention (Optimizing colorectal cancer screening through quality circles of primary care physicians, Bern, Switzerland 2021).

The proportion of patients previously tested or who planned to be tested did not differ significantly between the intervention and the control group (68% vs. 60%). Risk difference was 8%; OR was 1.37 (95% CI: 0.81 to 2.31, p 0.24) and OR 1.31 (95% CI: 0.69 to 2.48, p 0.40) adjusted for age, gender and location of the physicians and age and gender of patients. See Figure 3; Table 3 for details.

### Sensitivity analysis

In a sensitivity analysis restricted to the intervention group, the proportion of patients up to date with screening increased from 55% to 57.9% between the first and second data collection. Screening rate difference was 2.9% (95% CI:1.1%–6.9%); OR was 1.29 (95% CI:1.08 to 1.55, p < 0.01) adjusted on hierarchy of PCP and QC.

Three PCP participated in our trial providing patient screening data but did not provide information on their age, sex or practice location. We used mean substitution in our sensitivity analysis assuming a sex distribution of 50% male 50% female, with physicians aged 40–49 years and in an urban practice. To address missing outcome values, we applied multiple imputation using information on the sex and age of the relevant patients, resulting in a total of 181 missing screening statuses being imputed. With the mean substitution and imputed values, the OR was 1.71, (95% CI 1.02 to 2.89, p 0.04) controlled for the hierarchy of PCP and QC and adjusted for age, gender and location of the physicians and age and gender of patients.

## Discussion

### Summary

We found that the CRC screening rate in average-risk patients was 15.5% higher in the intervention group than in the control group. Our sensitivity analysis also showed that CRC screening rates significantly increased after the intervention when hierarchical models on the level of PCP and QC were applied. We found that the number of FOBT prescriptions, discussions of CRC screening during consultations and patients deciding to receive screening after the patient consultation did not increase significantly in the intervention group compared to the control group. However, we also found that the increase in CRC screening rate within the intervention group was 2.9%, a much lower percentage than the 15.5% in the main analyses, suggesting a potential selection bias of PCP collecting data in the intervention and control group. Thus, while CRC rates increased by 15.5% in the main analyses, we have low confidence that the magnitude of increase is only due to the intervention.

### Applicability of the results

To increase the possible uptake of our intervention, we collaborated with PCP to develop the intervention and data collection tools. To reduce barriers for PCP, we tested the feasibility of data collection and the suitability of all intervention materials in a pilot trial before this study began. Anticipating barriers should increase the applicability of our trial to PCP with heavy workloads. We expected all participants would provide a valid and accurate data set. The trial was only performed in the German-speaking part of Switzerland, with possible barriers in applicability to other language regions and to other countries with different healthcare structures as well as other socioeconomic levels.

### Comparison with existing literature

The CRC screening rate of 58% in our intervention group was higher than the rate we measured in earlier studies based on Swiss insurance claims data (48.4% and 42.5%) [23, 24] but far below the 70% we anticipated after our pilot study [8] and below the 62% rate suggested by our previous survey [5]. Our low screening rates may be because participating PCP did not discuss CRC screening with as many eligible patients or did not promote FOBT. We think it is likely that participating PCP did not actively promote FOBT; discussion rates and FOBT prescription rates were quite low. We know that PCP who prescribe more FOBT see an increase in their CRC screening rates [15]. Two international studies found CRC screening rates were as high as 68% [7] or 69% [10] when patients were given a choice between two screening tests. Our sensitivity analysis shows a smaller increase in screening rate in the intervention group of 2.9% than we expected in our sample size calculation and another similar trial produced with a shown screening rate increase of 9.3% in the intervention group through a quality circle based intervention [25].

Our intervention failed to convince PCP to promote FOBT as a valid option for CRC screening, perhaps because they continue to prefer colonoscopy. Barriers to promoting FOBT may also include an inability to devote time to promote FOBT during their practice as stated in a large literature review concerning barriers and facilitators in CRC screening [26]. Promoting FOBT in addition to promoting screening within a time-limited consultation needs time management, which poses a challenge for PCP.

Our intervention consisted of a multifaceted approach that included a basic training session, PCPs who gather daily data followed by individual performance feedback and discussions during three visits to QCs. This combination of multiple interdependent components and various activities in the social context of QCs are properties of a complex intervention [27]. Such interventions require careful planning, implementation, and evaluation due to their non-linear interactions and potential for variable outcomes, as seen in our study. We encourage future researchers to evaluate such complex trials using theory-driven approaches like realist inquiries, which necessitate the collection of qualitative data to explain the varying outcomes [28, 29].

### Limitations

Our decision to use de-identified patient data and rely on PCP self-reports meant PCPs did not need to obtain patient consent for data collecting, which reduced the risk of selection bias in the patients from whom PCPs retrieved data. However, this choice also meant that we could not verify the CRC testing rates reported by physicians, so we cannot rule out social desirability bias. As a result, we thus might have overestimated screening rates. But our estimates were only slightly higher than the 48.4% estimate from our earlier study in the general Swiss population [23].

We decided not to collect baseline screening uptake from patients in the control group (directly post-randomization), to prevent data collection from becoming an intervention (i.e., PCP inquiring about screening status could trigger testing). Consequently, our primary analysis compared screening uptake between groups at follow-up, not the change between baseline and follow-up. This design choice limited our ability to assess comparability between groups at baseline at the patient-level. A baseline imbalance in screening rates, or other source of residual confounding, could have caused us to overestimate intervention effect. Indeed, the screening rate was 55% in the intervention group prior to the intervention, compared to 42% in the control group, without any intervention. This issue reflects baseline non-comparability rather than selection bias. Selection or participation bias may instead arise from PCP recruitment and participation processes, which are discussed separately below.

As the randomization process occurred at the quality circle level, this resulted in a small number of clusters. With few clusters, randomization may fail to fully balance cluster-level characteristics between study arms, which may lead to baseline imbalance, residual confounding, and reduced precision of effect estimates.

The low participation rate and participants dropping out may restrict the generalizability of the findings, introducing a selection bias whereby the participating PCP in these QCs do not represent all PCP. This challenges the feasibility of implementing this intervention to enhance CRC screening uptake. Further research should explore other means of recruiting PCP in QCs, or the acceptability of our intervention outside of a research setting, or simpler intervention. Two QCs dropped out before we collected their baseline data and one dropped out before follow-up, all in 2020. This reduced the number of participants, and the uneven distribution between intervention and control limited our ability to confidently generalize our results to PCP. The 2020 dropouts were due to the pandemic, which forced some QCs to focus on more urgent healthcare tasks [30]. The pandemic also required us to extend our trial, causing us to deviate from our protocol, as seen in other studies [31]. Since there has been a trend of increased CRC screening in the general population over the last decade in Switzerland, extending the trial duration may have led us to observe natural trends in CRC screening rates. For example, the moderate increase in CRC screening rates in the intervention group might simply reflect this natural trend rather than the effect of the intervention.

### Implications

Our intervention did not successfully raise the FOBT screening rate, likely because it did not successfully persuade PCP to give patients an informed choice of screening options. But our intervention did show that performance feedback within a quality circle intervention is feasible and may *increase* the proportion of patients screened for colorectal cancer. Future interventions should identify the reasons for PCP reluctance to prescribe FOBT and explore possible ways to change this reluctance.

### Conclusion

A series of three training sessions, along with performance feedback, in QC of PCP significantly increased screening rates, highlighting their potential as effective tools for improving preventive care practices. However, we did not collect baseline screening rates for the control group. Given the low increase in screening rates in the intervention group, selection bias among participants in the intervention and control groups is a likely explanation for the findings. The observed differences may therefore not be due to the intervention. Given the lack of observed effect on FOBT screening, our results emphasize the need for interventions that effectively convince doctors of the efficacy of fecal occult blood tests in detecting colorectal cancer.
