# Supplementary material for: Optimizing colorectal cancer screening through quality circles of primary care physicians: a cluster randomized controlled trial
Source: Int J Public Health. 2026 May 20;71:1608462. doi: 10.3389/ijph.2026.1608462 (PMC13229839; doi:10.3389/ijph.2026.1608462)
Supplement: Supplementary file 1 [file Supplementaryfile1.docx]

Appendix:

# Survey among moderators:

# A survey asking for basic information on the quality circle, number of members, meeting frequency and structure, the moderator’s qualifications and if they receive any funding.


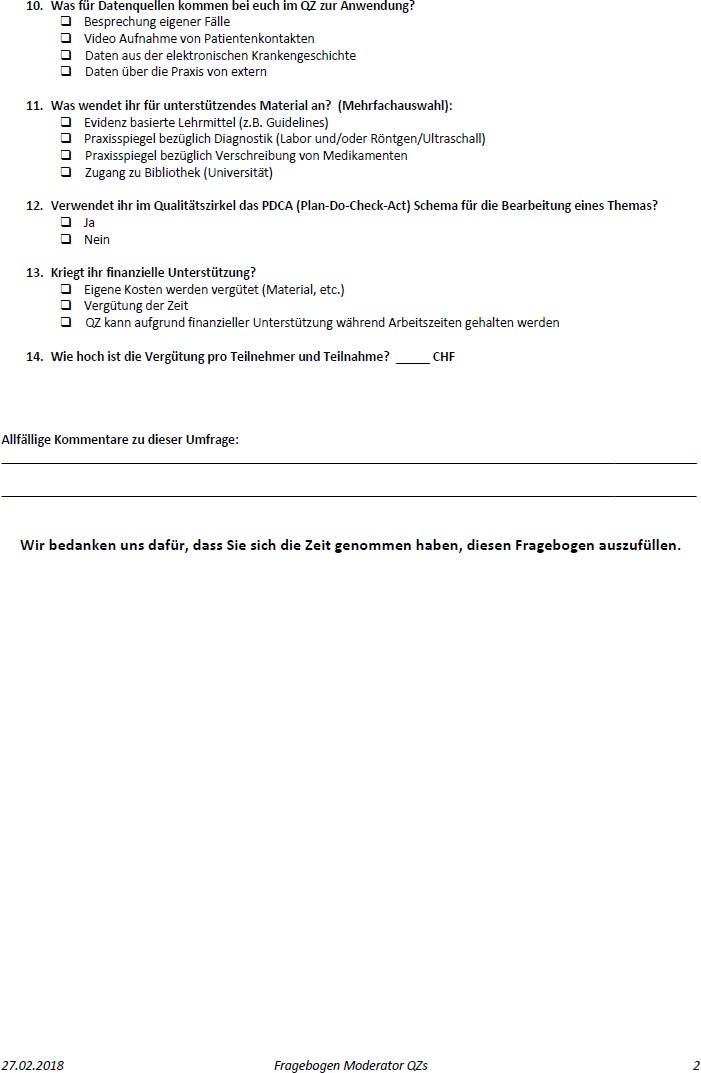

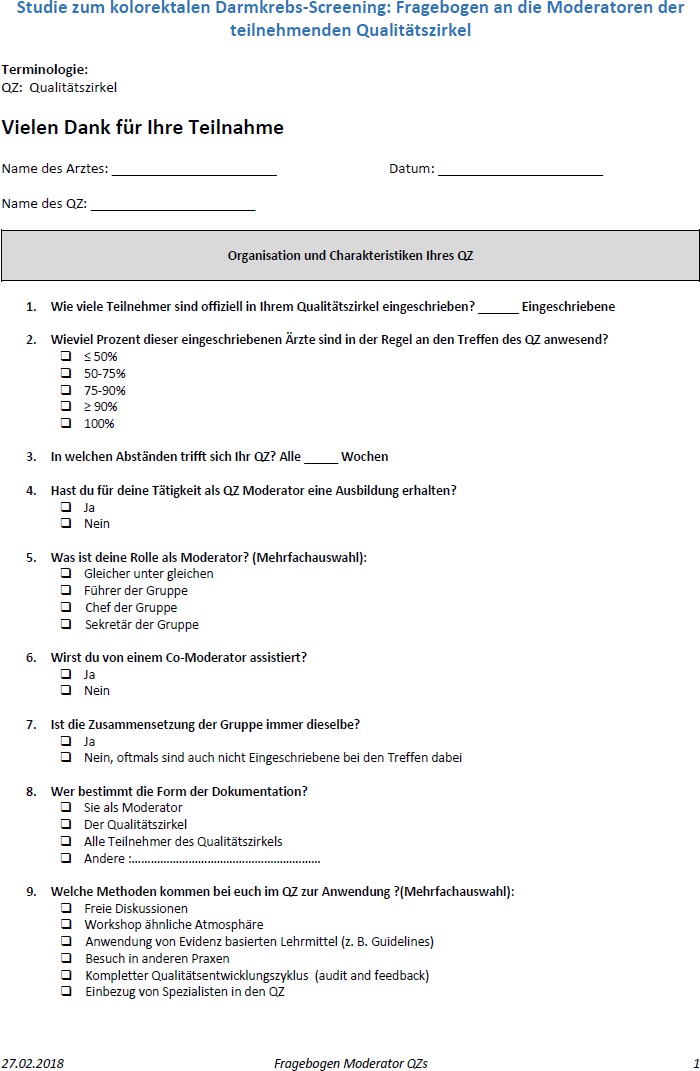


Timeline intervention and control:


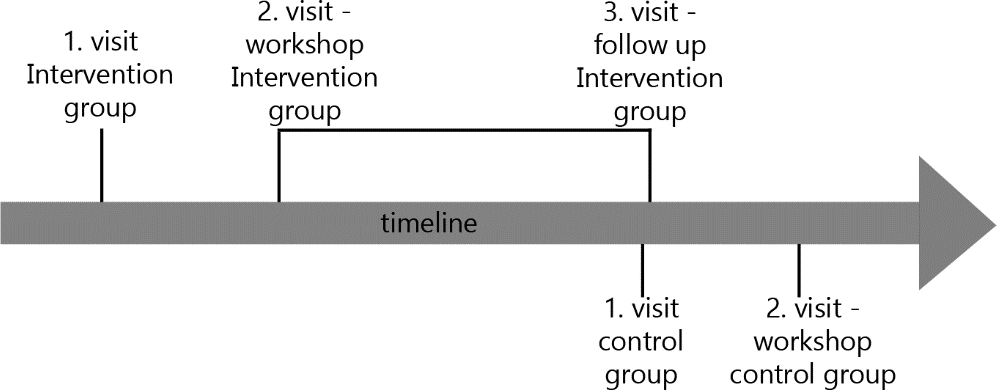


Baseline questionnaire:

This questionnaire begins with introductory information on key terminology related to colorectal cancer screening, including definitions of “screening” and the fecal occult blood test (FOBT). It also outlines the criteria for identifying asymptomatic patients at moderate risk for colorectal cancer and provides guidance on appropriate situations for informing patients about screening.

The main section explores primary care physicians’ (PCPs) attitudes and practices regarding screening recommendations for moderately at-risk patients. It includes questions on:

- Reasons for recommending or not recommending colorectal cancer screening

- The age at which PCPs typically begin recommending screening

- Estimated number of patients screened in the past six months within their practice

- Distribution of screening methods among patients who underwent screening

- Strategies used to explain screening procedures to patients

- Methods for reminding themselves to discuss screening during consultations

The final section collects baseline information about the participating physicians, including:

- Geographic location of their practice

- Age group of the respondent

- Practice characteristics (e.g., group vs. solo practice, available equipment, patient data management systems, and use of performance metrics)


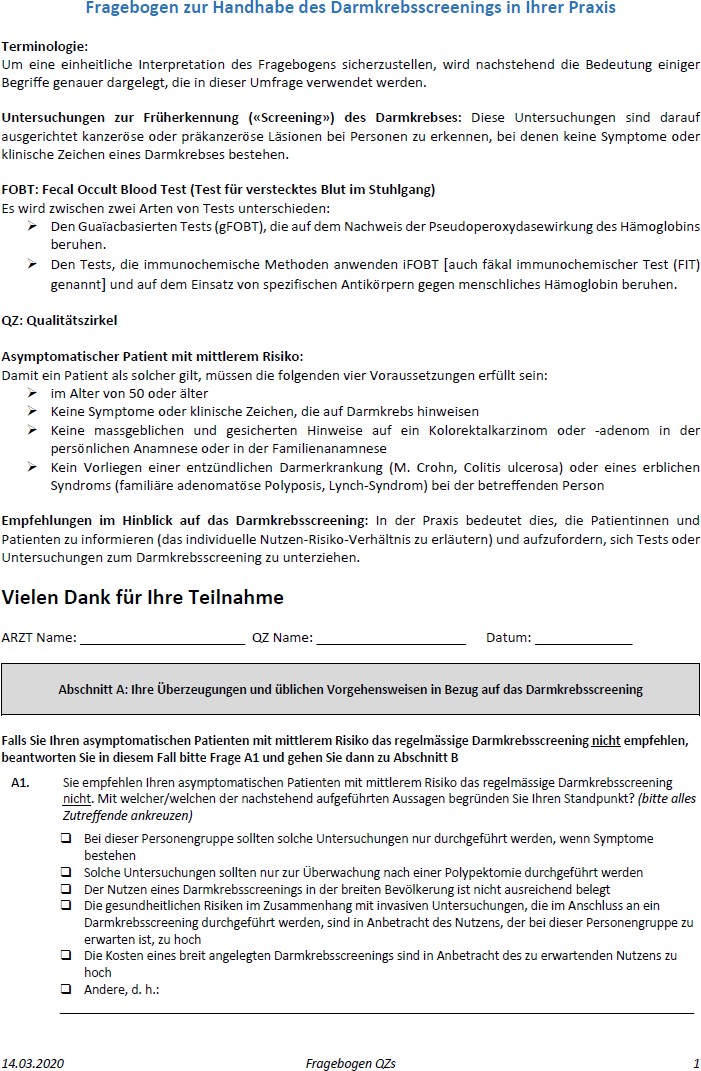

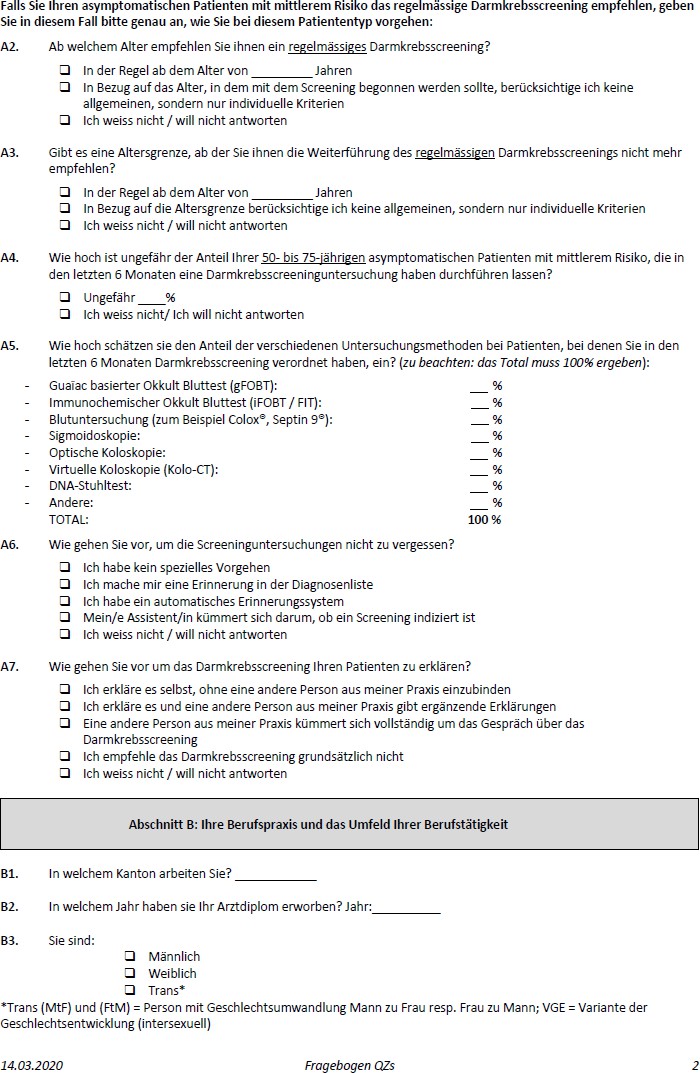


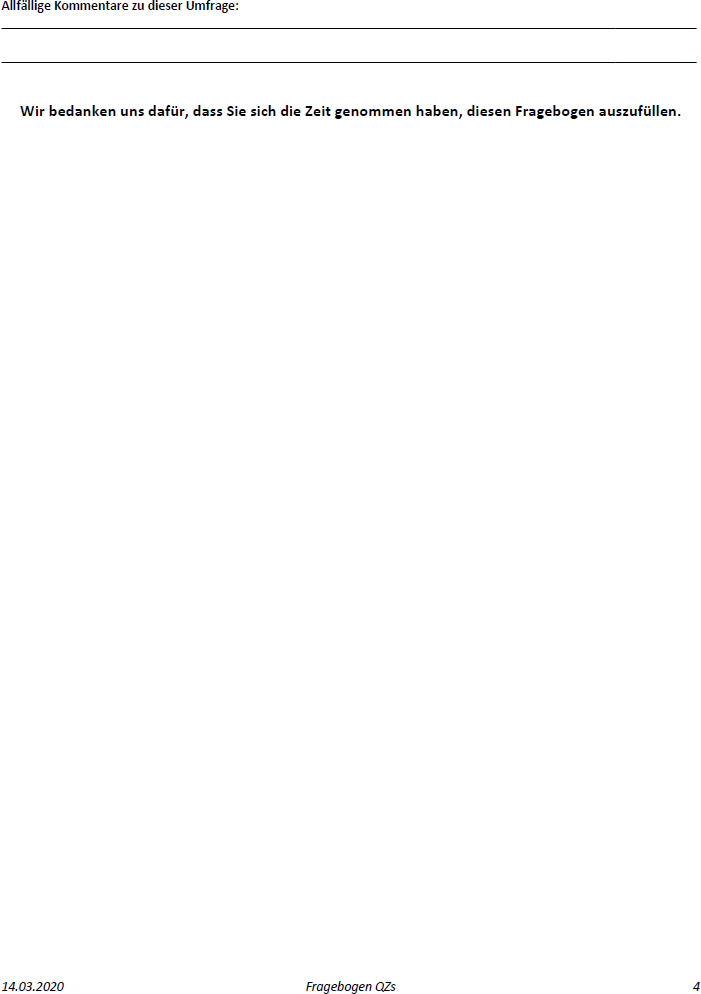

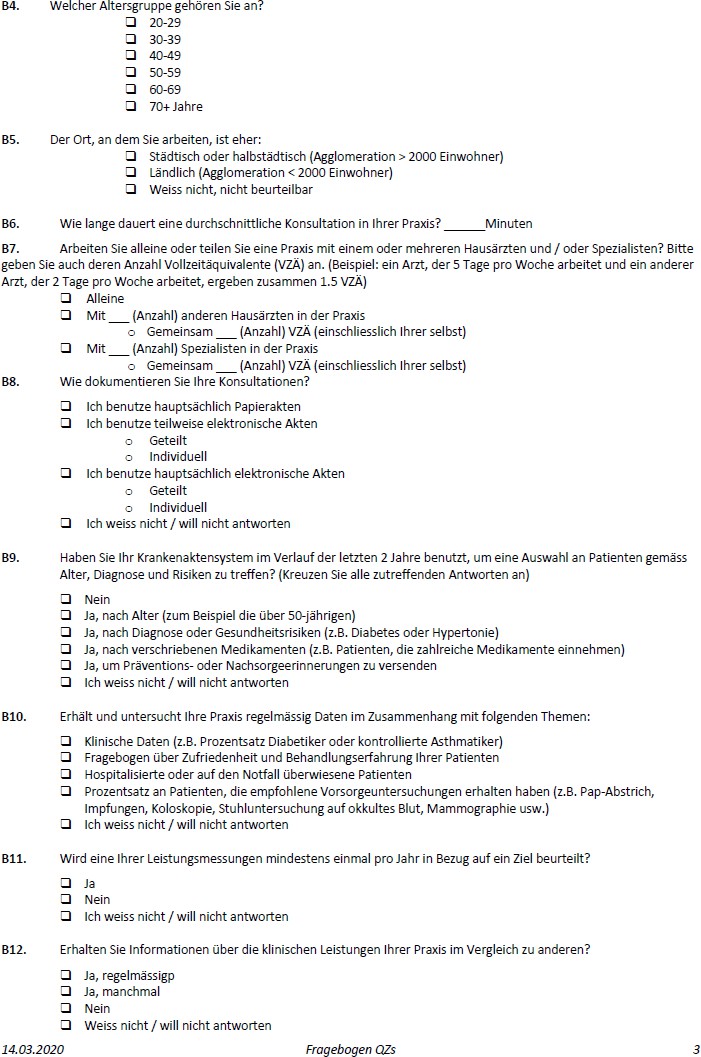


Overview of Colorectal Cancer Screening Materials

A two-page summary of current guidelines and evidence related to colorectal cancer screening in Switzerland.

The content is organized as follows:

- Epidemiological Context

Presents recent data on the incidence and prevalence of colorectal cancer in the Swiss population, with emphasis on age-related risk and screening relevance.

- Diagnostic Accuracy of Screening Methods

Flowcharts illustrate the sensitivity and specificity of fecal immunochemical testing (FIT) and colonoscopy, including total numbers where available. These visual aids support understanding of test performance and decision-making thresholds.

- Screening Pathways and Follow-Up

A schematic overview outlines recommended follow-up procedures based on initial screening results, aligned with national and international guidelines.

- Risk–Benefit Profiles

Comparative tables summarize the potential benefits and risks associated with FIT and colonoscopy. Each assessment is referenced to its underlying evidence base, including peer-reviewed studies and official recommendations.


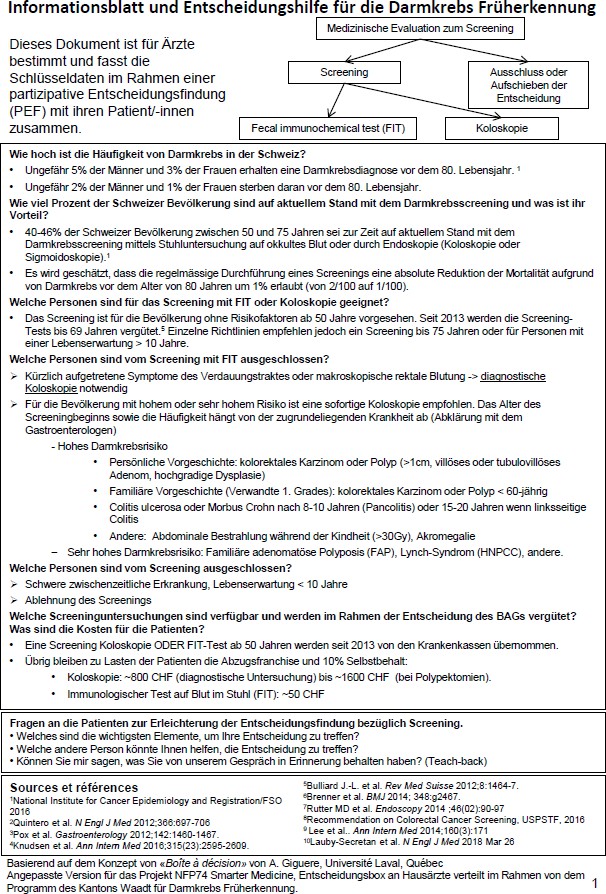

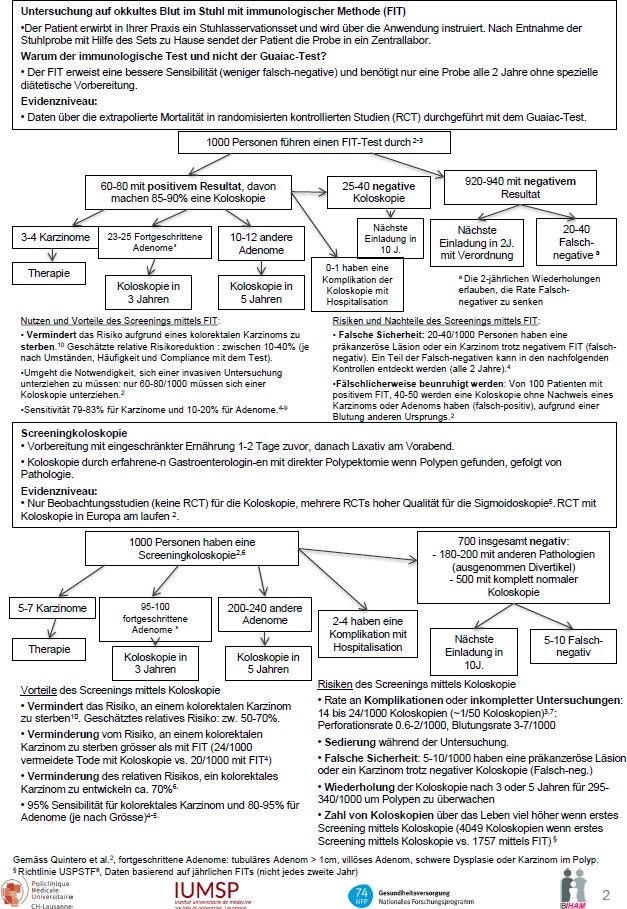


# Decision Board:

Two laminated sheets provide visually engaging, evidence-based information designed to support primary care providers in discussing colorectal cancer screening with patients. The materials include clear explanations of the prevalence and mortality rates of colorectal cancer in Switzerland, along with accessible illustrations showing where and how the disease typically develops in the body. They describe the two guideline-recommended screening methods—fecal occult blood test (FOBT) and colonoscopy—detailing how each procedure is conducted. The sheets conclude with a comparative table that outlines both screening options in terms of cost, associated risks and benefits, and diagnostic accuracy, with all data grounded in current clinical guidelines and research evidence.


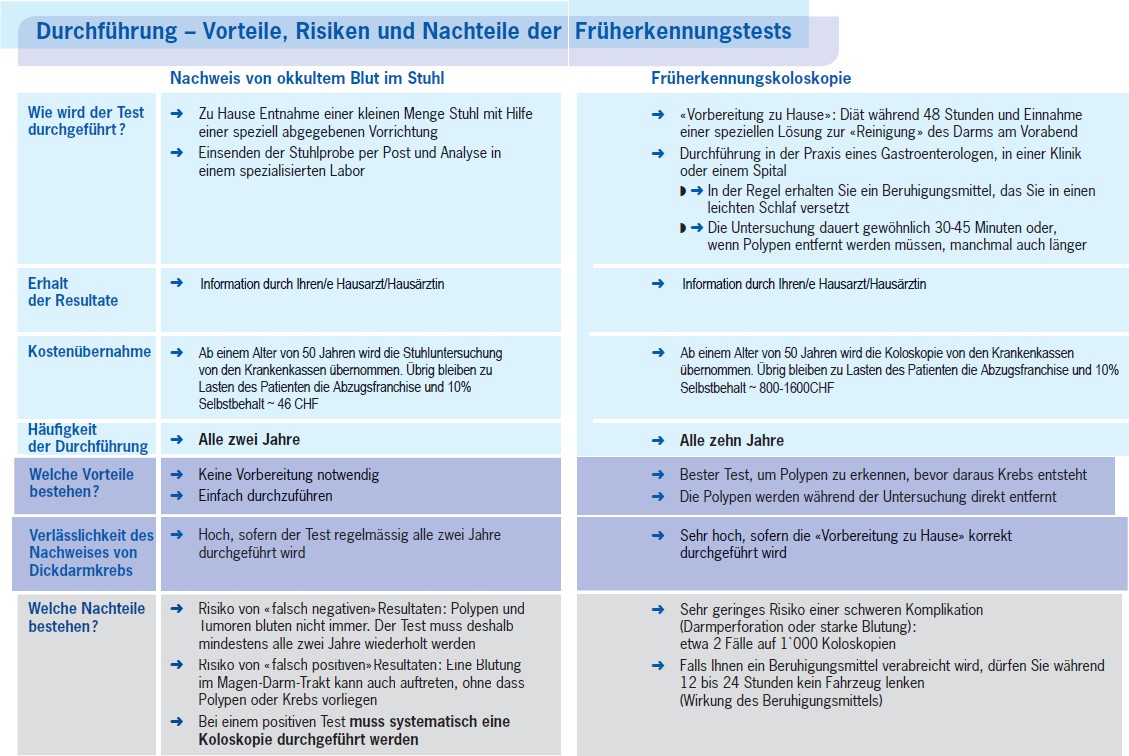

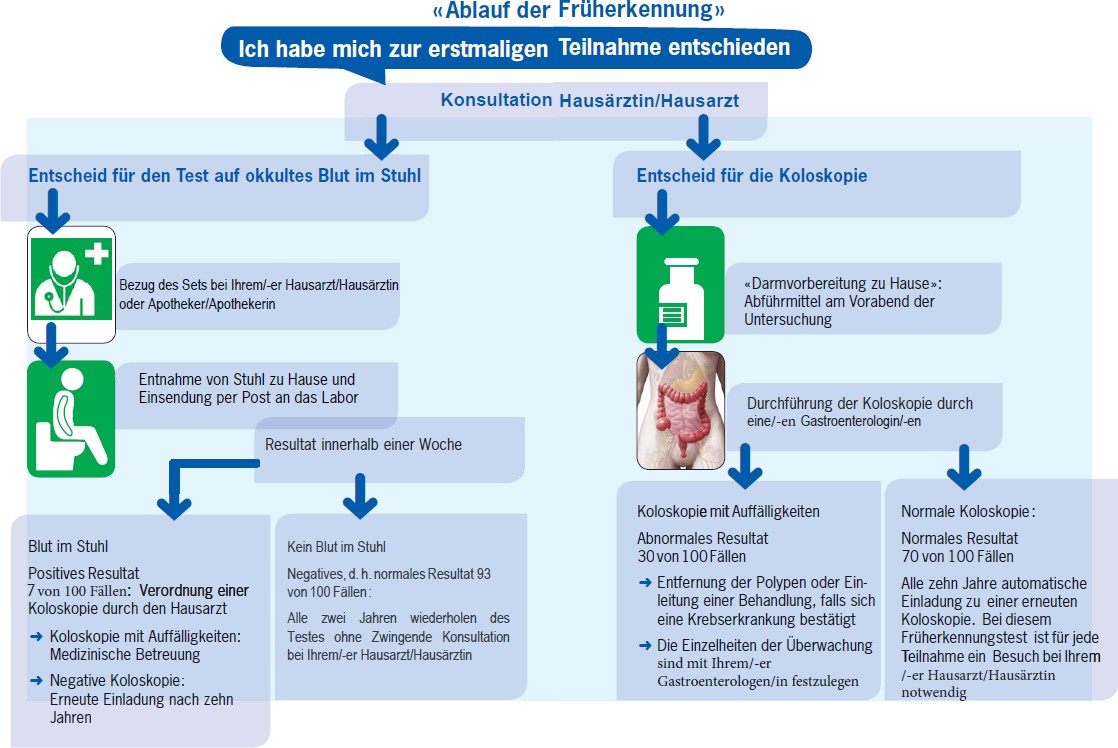


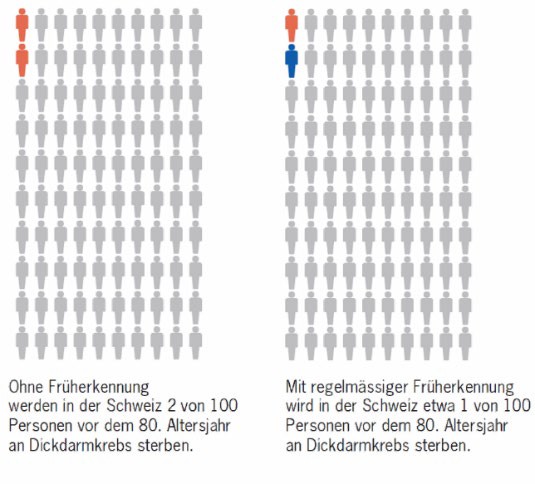

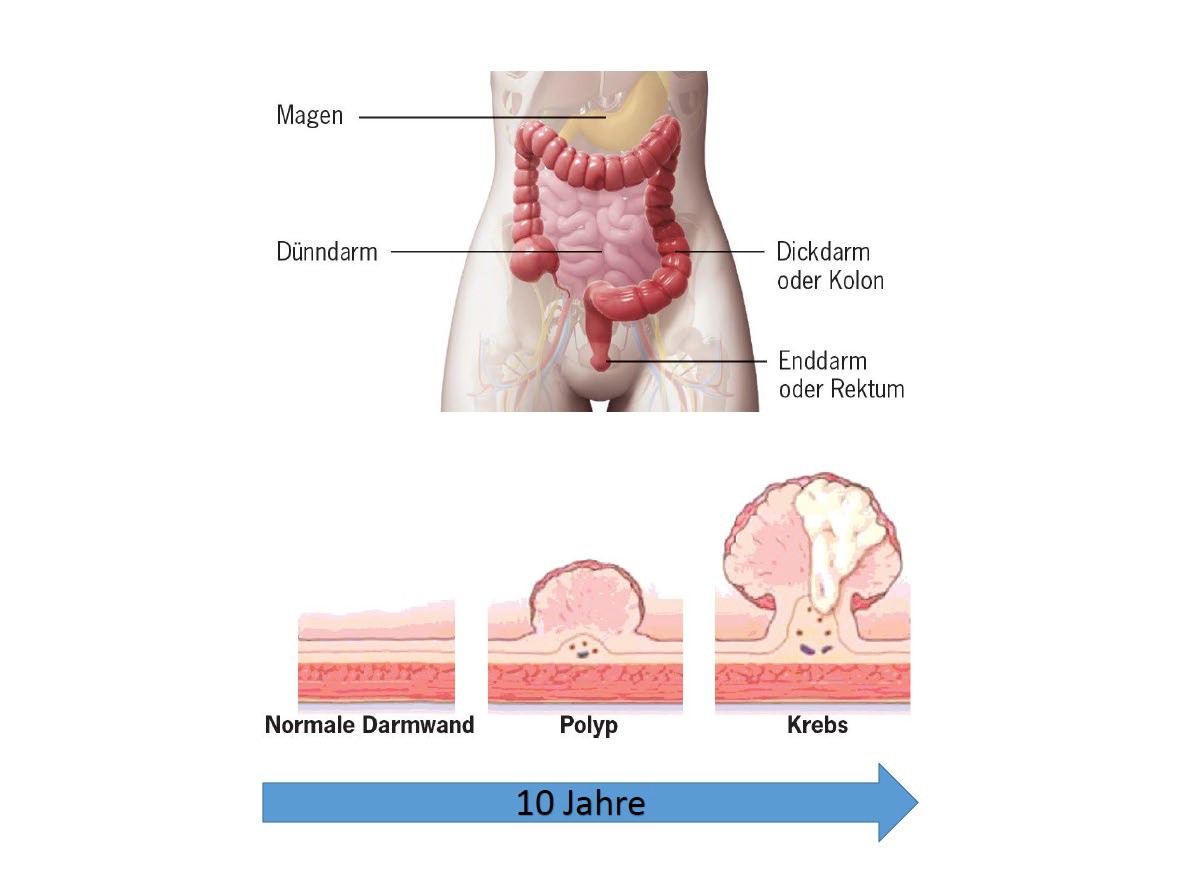


# Data collection form:


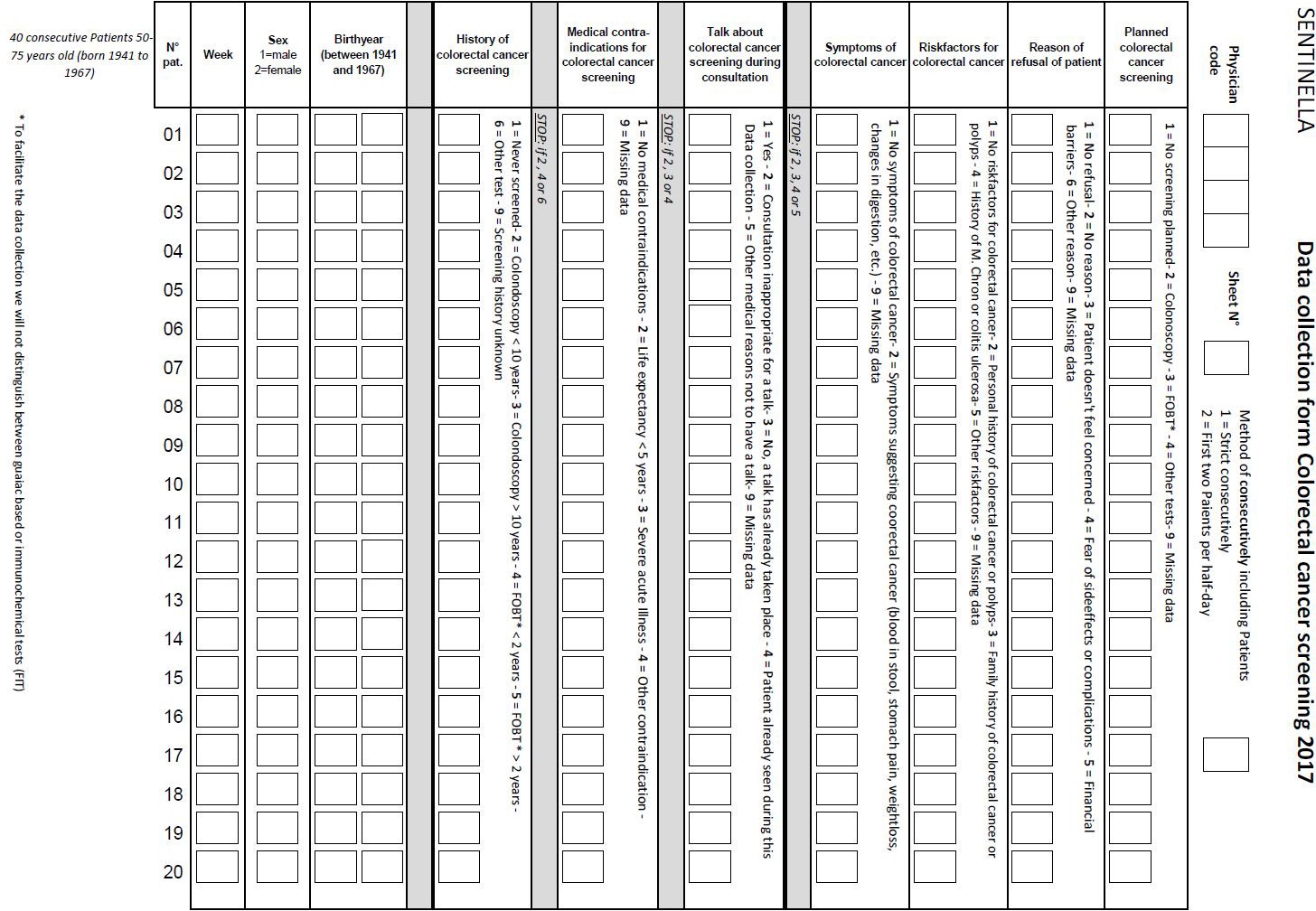


Flowchart of data collection process:

This flowchart illustrates the structure of the data collection form, detailing how specific participant responses determine the progression or termination of data collection. It visually maps the decision points within the form, indicating which answers prompt additional questions and which lead to the conclusion of the data entry process.


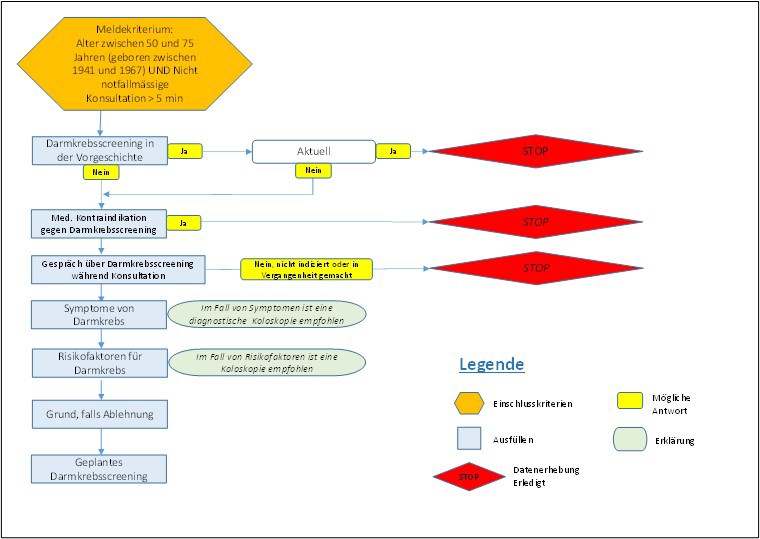


Data collection overview:

This three-page document provides a comprehensive explanation of the data collection process. It outlines the purpose of data collection within the study, offers detailed instructions on how to complete the data collection form, and includes a flowchart illustrating the logic of the form—specifically, which responses trigger additional questions and which lead to the conclusion of data entry.

The document also contains a complete list of all variables collected through the form, each accompanied by a definition and explanation. For example, the variable “sex” refers to the patient’s self-identified sex, coded as follows: 1 = man, 2 = woman, 3 = trans. In this context, “trans” is defined as individuals who have undergone a gender transition from male to female or female to male.

At the end of the document, contact details are provided for the research assistant responsible for data coordination, including their name, email address, telephone number, and institutional affiliation, should further clarification or support be required.
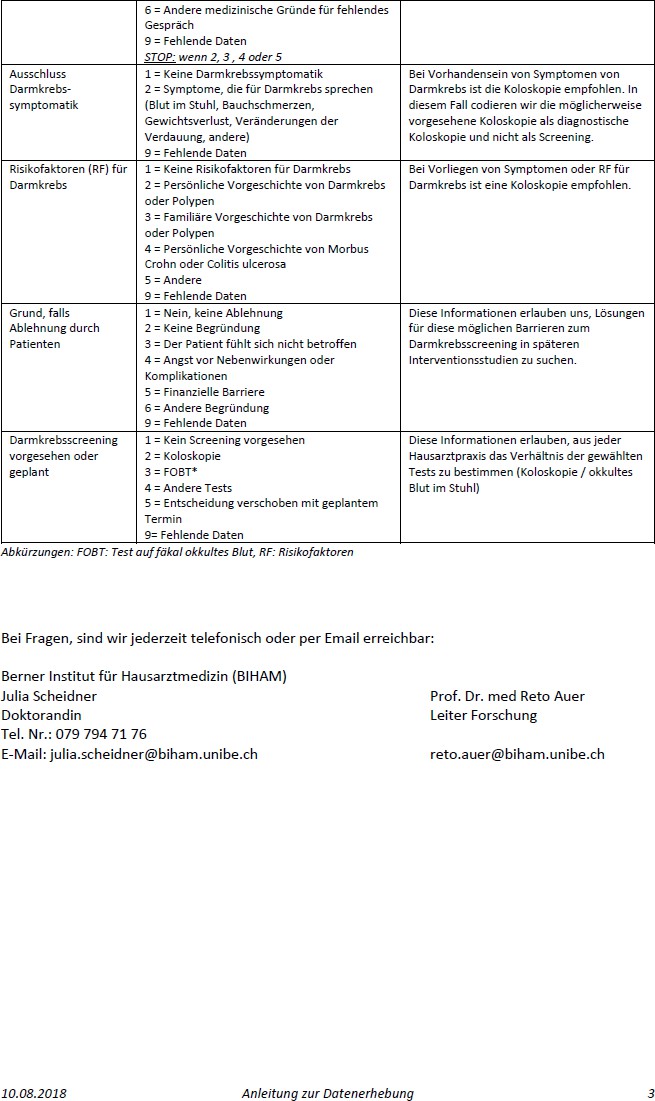

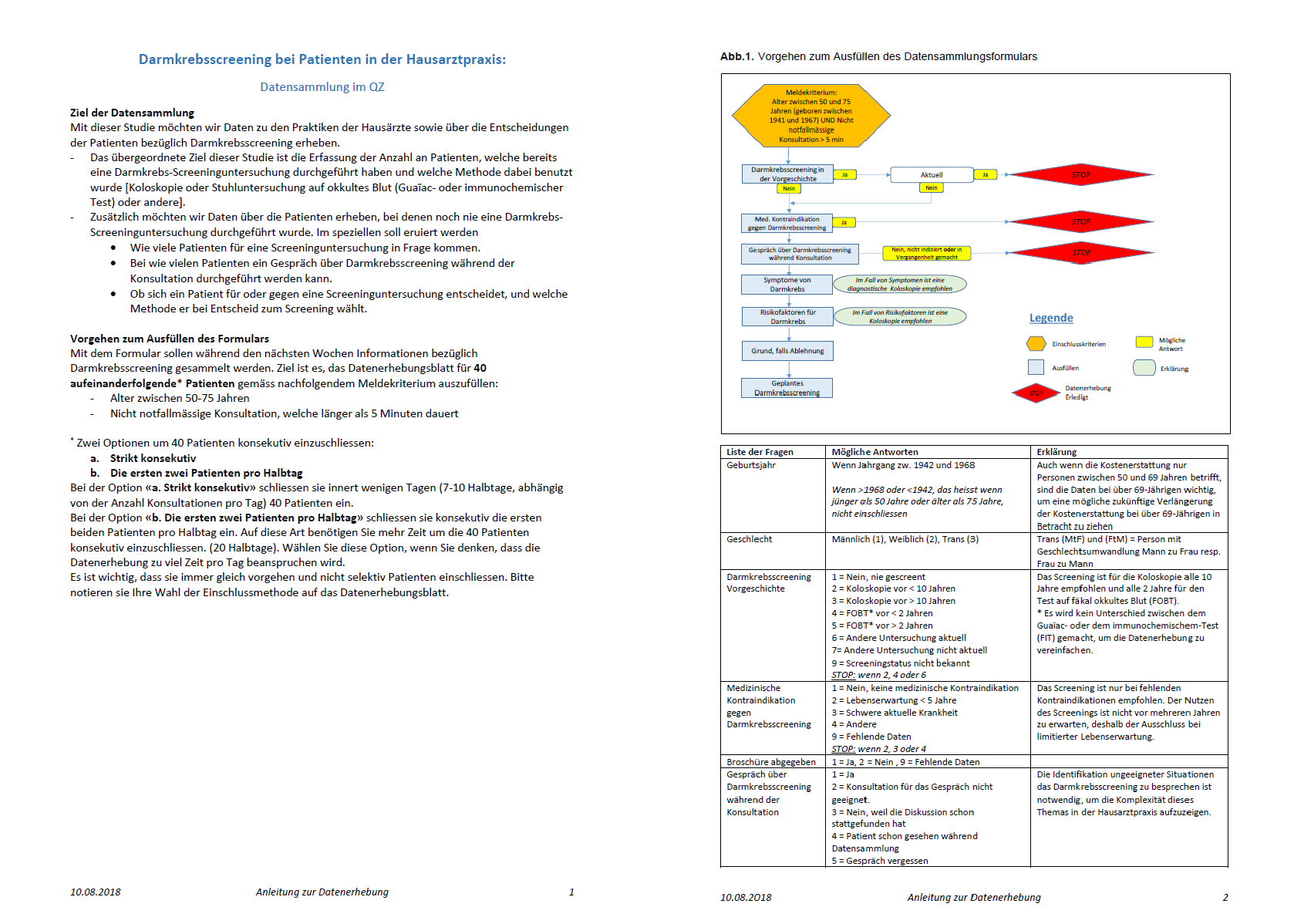


Example of individual performance feedback for PCP of a QC:


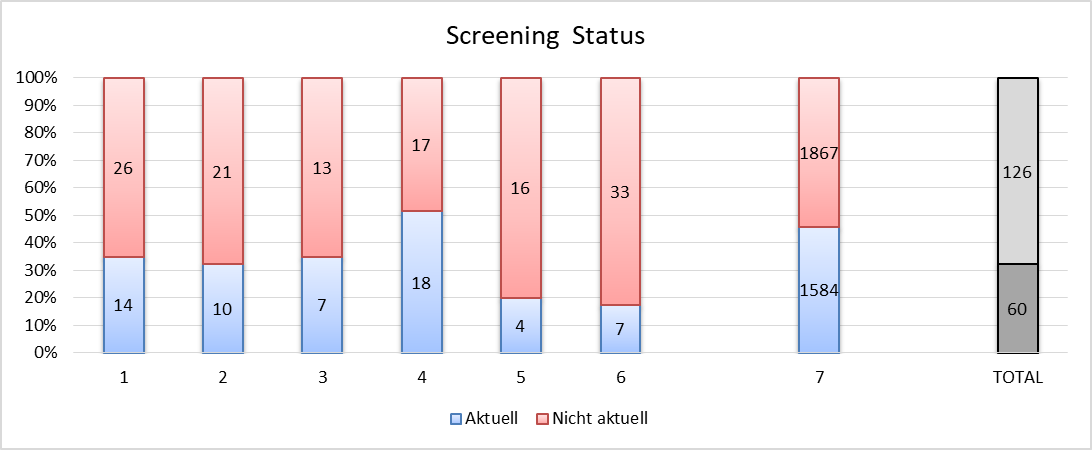

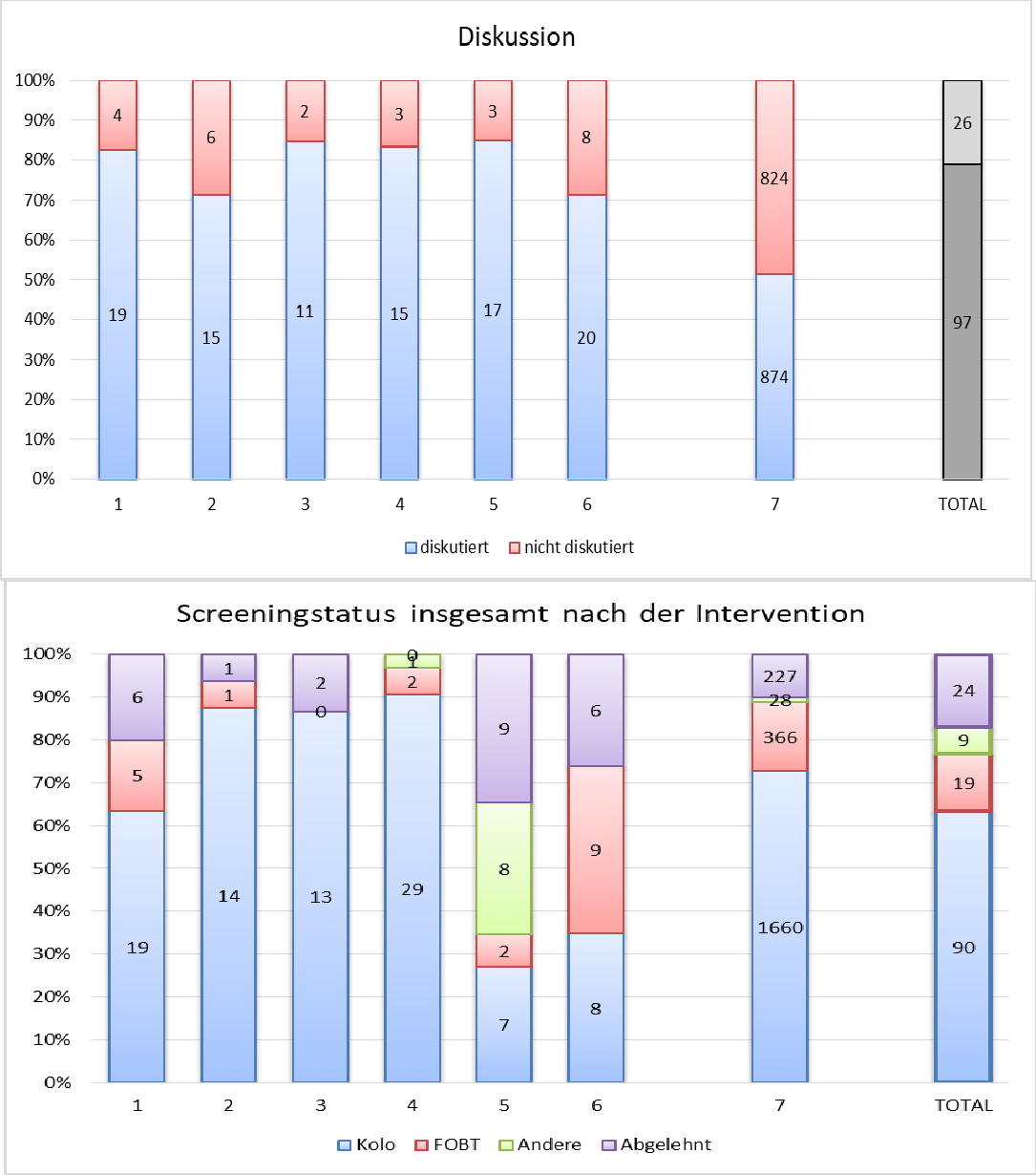

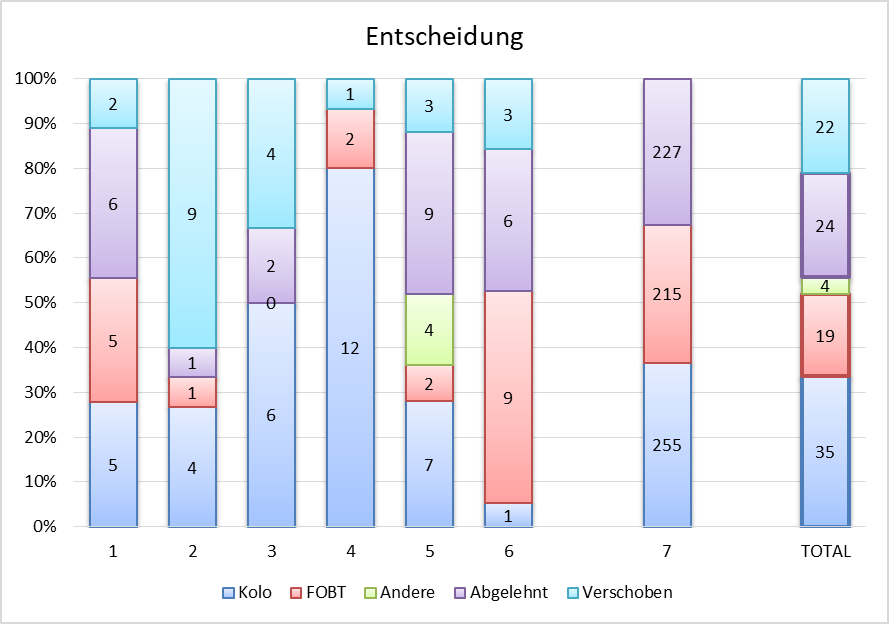


* Each bar represents an individual PCP and the total number of patients for whom they provided data. The bar at the very right of each section is the total number of patients for whom the QC collected data. "Screening status" shows the proportion of screened patients versus not screened patients per PCP, "Diskussion" shows with which of the not screened patients had a screening discussed, "Entscheidung" shows which decision the patients chose after the discussion and "screeningstatus insgesamt nach intervention" shows the proportion of patients who were screened or decided to be screened through colonoscopy, FOBT, other or not to be screened.
